# Interplay of Lymphocytes with the Intestinal Microbiota in Children with Nonalcoholic Fatty Liver Disease

**DOI:** 10.3390/nu14214641

**Published:** 2022-11-03

**Authors:** Tian Liang, Dan Li, Jiawulan Zunong, Menglong Li, Nubiya Amaerjiang, Huidi Xiao, Nourhan M. Khattab, Sten H. Vermund, Yifei Hu

**Affiliations:** 1 Department of Child, Adolescent Health and Maternal Care, School of Public Health, Capital Medical University, Beijing 100069, China; 2 Yale School of Public Health, Yale University, New Haven, CT 06510-3201, USA

**Keywords:** gut microbiota, dysbiosis, children with NAFLD, lymphocytes, microbial network

## Abstract

Abnormally high lymphocyte counts are seen in persons with nonalcoholic fatty liver disease (NAFLD). Gut microbiota dysbiosis is a risk factor for NAFLD. We assessed the gut microbiota of 63 healthy children and 63 children with NAFLD using 16S rRNA gene and metagenomic sequencing to explore the relationships. Compared with healthy children (HC group), the Bacteroidetes, Verrucomicrobia, and *Akkermansia* were less abundant, while the Actinobacteria were more abundant in children with NAFLD (FLD group). To understand the effect of lymphocytes on the gut microbiota of children with NAFLD, we compared the microbiota of 41 children with NAFLD and high numbers of lymphocytes (FLD_HL group) and 22 children with NAFLD and low numbers of lymphocytes (FLD_LL group). The abundances of Bacteroidetes, Verrucobacterium, and *Akkermansia* increased and Actinobacteria decreased in the FLD_LL group compared to the FLD_HL group. *Akkermansia* was negatively correlated with lymphocyte count. NAFLD may disturb the gut microbiota in children through reducing the abundance of *Akkermansia* and increasing the abundance of proinflammatory bacteria, such as *Escherichia-Shigella*. Conclusions: High lymphocyte counts are associated with disturbances of gut microbiota and emergence of opportunistic pathogens in children with NAFLD.

## 1. Introduction

Nonalcoholic fatty liver disease (NAFLD), recently named metabolic (dysfunction)-associated fatty liver disease (MAFLD) [1], is characterized by lipid accumulation and progressive steatosis in hepatocytes [2]. It is the most common chronic liver diseases among children and adolescents and is occasionally even seen in young children [3,4]. The prevalence of NAFLD in children was 33.78% from 2000–2021 [5]. It has been shown that NAFLD has a multifactorial etiology with factors like sex, a lack of exercise, maternal obesity, and use of antibiotics contributing to dysbiosis of the gut microbiota [2,3,4]. Unhealthy eating habits (high fat/carbohydrate/calorie diets) are major contributing factors to obesity and NAFLD [6].

There is a close bidirectional relationship between NAFLD and gut microbiota [7]. NAFLD causes gut microbiota imbalance, increased gut permeability, and activation of the Toll-like receptor 4 pathway, leading to further deterioration of NAFLD [8,9]. When compared with the microbiota of appropriate control subjects, the microbiota of NAFLD-associated persons often manifest decreased or altered phylogenetic diversity and significant differences in the abundance of bacteria [4,10]. Children with NAFLD have dysregulated glucose metabolism, lipid metabolism, and water–electrolyte metabolism, mitochondrial dysfunction, and increased oxidative stress compared to healthy children [4,10]. These metabolic pathways are closely related to the gut microbiota, but the specific mechanism via which NAFLD leads to alterations in the intestinal microbiota is unknown.

Gut microbiota contribute to the development and regulation of the gut mucosal immune system, as well as in obesity and NAFLD [1]. Short-chain fatty acids are a key metabolite produced by the intestinal microbiota to protect against NAFLD [11]. Short-chain fatty acids reduce hepatic lipid accumulation and improve liver function in mice [11]. Probiotics can be used for the intervention and treatment of NAFLD disease and can inhibit the progression of NAFLD through the lipopolysaccharides/Toll-like receptor 4 signaling pathway [12].

Gut microbiota may affect the progress of NAFLD. Dysbiosis of gut microbiota may disrupt the homeostasis of bile acid metabolism, affecting the organism’s hepatic lipid metabolism and glucose metabolism and inducing NAFLD [7,13]. Dysbiosis of the gut microbiota led to disruption of the gut–liver barrier and increased portal transport of bacterial endotoxins to the liver, promoting NAFLD progress [7]. On the other hand, a high-fat diet did not cause obesity and NAFLD in germ-free mice [14].

Lymphocytes are mainly composed of natural killer, T, and B cells. Natural killer cells induce inflammation by releasing proinflammatory cytokines and reactive oxygen species [15]. The massive infiltration of T lymphocytes and B lymphocytes exacerbates liver damage and inflammation [16,17]. Previous studies posited an interactive relationship among NAFLD, gut microbiota, and lymphocytes [1,18,19], but few studies have explored associations of lymphocyte levels with the gut microbiota of children with NAFLD. We sought to explore associations of lymphocyte counts and characteristics of gut microbiota in children living with or without NAFLD. Revealing the association among NAFLD, gut microbiota, and lymphocytes may inform interventions for children with NAFLD.

## 2. Materials and Methods

### 2.1. Study Subject Recruitment

In the present study, we enrolled 63 eligible children with NAFLD and matched 63 healthy control children from the Beijing Child Growth and Health Cohort (PROC) study in urban area of Shunyi District [20]. The inclusion criteria for cases were children aged 6–7 years old with ultrasonography-proven NAFLD and, for controls, a healthy liver and no other hepatic disease such as chronic hepatitis. The exclusion criteria for the study were being mentally retarded or having congenital cardiopulmonary insufficiency and/or other chronic diseases. Liver ultrasonography was performed by a certified physician using a Canon Aplio500 (Canon Medical Systems Co., Ltd., Tochigi, Japan) with a multifrequency convex transducer probe (4–11 MHz) to investigate the presence of NAFLD with hyperechoic texture or a bright liver standard (Abdominal ultrasound for the diagnosis of NAFLD). All participants provided blood and fecal samples for testing and had not taken antibiotics in the last month.

The study protocol was reviewed and approved by the Ethics Committee of Capital Medical University (No. 2018SY82) and complied with the Declaration of Helsinki and its subsequent revised ethical principles and comparable international ethical standards. The trial was registered with the China Clinical Trials Registry (ChiCTR) (www.chictr.org.cn/enIndex.aspx, No. ChiCTR2100044027, accessed on 2 November 2022). Reporting of results was performed according to the criteria described in the Consolidated Standards for Reporting Trials and the CONSORT extension for nonpharmacological trials (www.consort-statement.org, accessed on 2 November 2022).

### 2.2. Data Collection

We collected demographic and clinical data, including anthropometric and body composition measurements. These included research-caliber height and weight measurements, waist, hip, visceral fat area (VFA), percentage of body fat (PBF), and body mass index (BMI). Anthropometric and body composition measurements were conducted among children after overnight fasting and wearing light clothes with barefoot in the morning. Standing height was measured using a mechanical height meter (Zhenghe Medical Supply Manufacturer, Hengshui, China). Weight, WHR, VFA, and PBF were measured using a bioelectrical impedance body composition analyzer (multifrequency and multi-section contact eight-electrode analyzer H-Key 350, Beijing Seehigher Technology Co., Ltd., Beijing, China). BMI was calculated as weight in kilograms divided by height in meters squared (kg/m^2^).

Fasting blood samples were collected and sent for immediate testing. Lipid metabolism indicators such as high-density lipoprotein cholesterol (HDL-C), low-density lipoprotein cholesterol (LDL-C), and triglyceride (TG) were assayed by US AU5800^®^ automatic biochemical analyzer (Beckman Coulter Commercial Enterprise Co., Ltd., Shizuoka, Japan). Complete blood counts (automatic blood cell analyzer, XS-500i, Sysmex Co., Kobe, Japan) included white blood cell count (WBC), red blood cell count (RBC), hemoglobin (HGB), hematocrit (HCT), mean corpuscular volume (MCV), lymphocyte count (LYM), monocyte count (MONO), neutrophil count (NEUT), eosinophil count (EO), basophil count (BA), and immature granulocyte count (IG). 

Serum hepatic function panels (Hitachi LABOSPECT 008AS, Hitachi High-Tech Co., Tokyo, Japan) included alanine aminotransferase (ALT), alkaline phosphatase (ALP), cholinesterase (CHE), γ-glutamyl transpeptidase (GGT), prealbumin (PA), total protein (TP), and globulin (GLB). Fecal samples (0.5 g per tablet) were collected using a fecal occult blood card (BA-2020B, Baso diagnostics Inc., Zhuhai, China) and stored in a −80 °C freezer.

### 2.3. 16S rRNA Gene Sequencing

The QIAamp DNA Stool Mini Kit (QIAGEN, Hilden, Germany) was used for microbial DNA extraction. The Qubit 2.0 fluorometer (Thermo Scientific, Waltham, MA, USA) was used to detect the concentration and purity of bacterial DNA. Universal 341F (5′–CCTACGGGNGGCWGCAG–3′) and 805R (5′–GACTACHVGGGTATCTAATCC–3′) primers were used to amplify the V3–V4 region of the 16S rRNA gene. PCR products were separated by 2% agarose gel electrophoresis, purified using AMPure XP beads. Based on the Illumina protocol, DNA library was constructed from purified amplicons and sequenced on the Illumina Hiseq platform. FLASH was used for quality filtering and merging raw FASTQ files [21]. Chimeric sequences were detected and removed by UCHIME [22]. UCLUST (http://drive5.com/uclust/, accessed on 2 November 2022) was used to cluster operational taxonomic units (OTUs) based on a 97% similarity cutoff [23]. The USEARCH (http://drive5.com/usearch/, accessed on 2 November 2022) performed taxonomic annotation based on the Silva database (Release 128).

### 2.4. Metagenomic Sequencing

To further validate the 16S rRNA gene sequencing results, we performed metagenomic sequencing of fecal samples from 13 children (five healthy children, eight children with NAFLD) in this study. The metagenomics library was constructed using the Kapa DNA Hyper Prep Kit (Kapa Biosystems, Wilmington, MA, USA) and sequenced on the Illumina Hiseq platform. Raw reads were processed using the Trimmomatic software (human reads, adapter, low-quality sequences) [24]. Taxonomy was annotated via MetaPhlAn2 (http://huttenhower.sph.harvard.edu/metaphlan2/, accessed on 2 November 2022) [25]. The assembly of metagenomes was carried out using SOAP denovo (http://soap.genomics.org.cn/, accessed on 2 November 2022) [26]. MetaGeneMark software was used for gene prediction of assembled sequences [27]. All redundant sequences were removed with the aid of CD-HIT software (weizhongli-lab.org/cd-hit/, accessed on 2 November 2022) [28]. Gene abundance was computed using BBmap (https://sourceforge.net/projects/bbmap/, accessed on 2 November 2022). 

### 2.5. Bioinformatics and Statistical Analyses

We used the Vegan R package to analyze the alpha and beta diversity. Observed species, Shannon, Simpson, and evenness indices were used for alpha diversity analysis, for both richness and evenness. For beta diversity, we performed principal coordinate analysis (PCoA) and permutational multivariate analysis of variance (PERMANOVA) using Bray–Curtis distance metrics [29]. We used linear discriminant analysis [30] effect size (LEfSe) methods to identify microbial taxa that differed significantly between groups (LDA score ≥ 2) [31]. Details of our microbial network construction methods were described previously [32].

Demographic statistics and body composition measurements of the case and control groups were presented as mean ± standard deviation (SD). We performed Chi-square tests, independent *t*-tests, and Wilcoxon rank-sum tests to determine the significance of differences in groups. Pearson correlation analysis was performed to calculate the correlation coefficients between clinical indicators and the relative abundance of microbial genera. *p*-Values were adjusted using the false discovery rate (FDR), and a two-tailed *p*-FDR of 0.05 was used to assess statistical significance. The raw sequence data were deposited in the Genome Sequence Archive in Beijing Institute of Genomics Data Center with accession numbers CRA007292 and CRA007303. The shared URL is http://bigd.big.ac.cn, accessed on 2 November 2022.

## 3. Results

### 3.1. NAFLD Alters the Composition of Gut Microbiota in Children

The 126 children were aged 6.7 years. The clinical measures in the 63 children with NAFLD were higher than those 63 healthy children, including height, weight, waist, hip, PBF, VFA, BMI, WBC, RBC, HGB, HCT, MONO, NEUT, IG, TG, ALP, CHE, GLB, TP, ALT, PA, and GGT, consistent with the NAFLD clinical expectations of these indicators. HDL-C was higher in healthy children (Table 1 and Supplementary Figure S1). 

**Figure 1:**
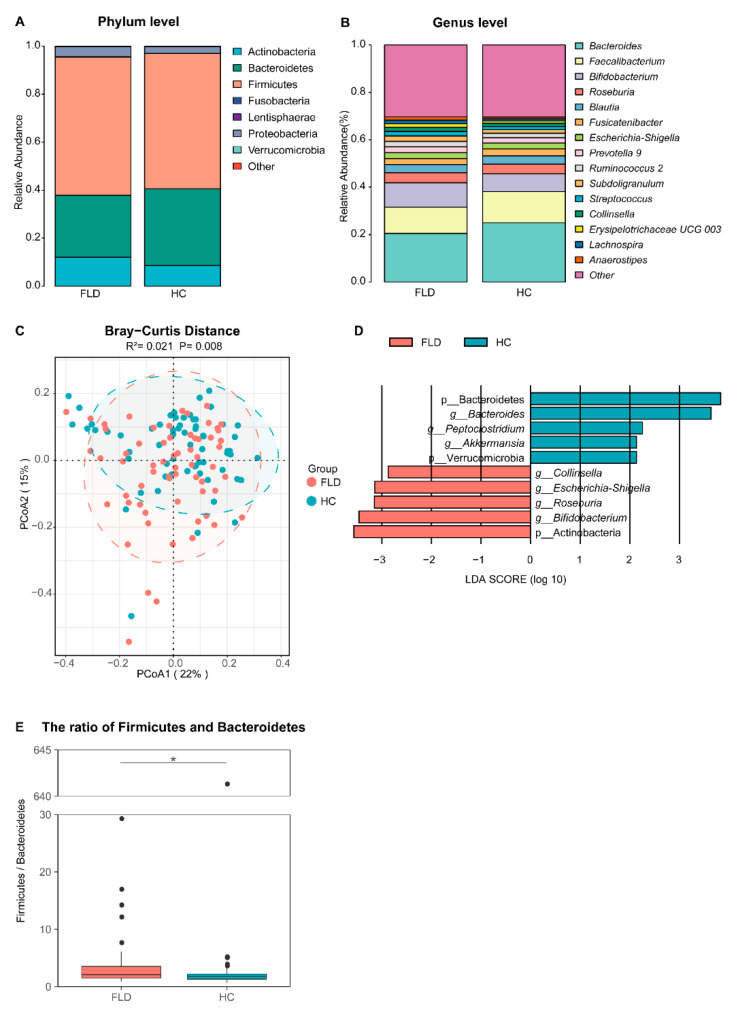
Relative abundance of gut microbiota at the phylum level (**A**) and the genus level (**B**). (**C**) The beta diversity of microbial communities between the children with NAFLD and healthy children was significantly different (R^2^ = 0.021, *p* = 0.008). (**D**) The LEfSe results of the children with NAFLD and healthy children. High abundances of Actinobacteria, *Collinsella*, *Escherichia-Shigella*, *Roseburia*, and *Bifidobacterium* were found in the gut microbiota of children with NAFLD. The abundances of Bacteroidetes, Verrucomicrobia, *Bacteroides*, *Peptoclostridium*, and *Akkermansia* were higher in the gut microbiota of healthy children. (**E**) The ratio of Firmicutes and Bacteroidetes was significantly higher in gut microbiota in healthy children than in children with NAFLD, * *p* < 0.05.

The saturated rarefaction curves and rich rank abundance curves showed sufficient sequencing depth and comprehensive sampling in this study (Supplementary Figure S2A,B). Following quality control, we obtained a total of 9,252,106 sequences, with an average of 73,429 sequences per sample. The bacterial phyla represented in the gut microbiota of children included Firmicutes (56.97%), Bacteroidetes (28.86%), Actinobacteria (10.36%), Proteobacteria (3.5%), Verrucomicrobia (0.21%), Fusobacteria (0.07%), and Lentisphaerae (0.01%) (Figure 1A). Additionally, all genera of *Bacteroides* (22.65%), *Faecalibacterium* (12.23%), *Bifidobacterium* (8.89%), *Blautia* (3.80%), *Roseburia* (3.55%), *Prevotella 9* (2.93%), *Fusicatenibacter* (2.53%), *Ruminococcus 2* (2.27%), *Subdoligranulum* (2.11%), *Escherichia-Shigella* (1.63%), *Streptococcus* (1.61%), *Lachnospira* (1.46%), *Anaerostipes* (1.42%), *Erysipelotrichaceae UCG 003* (1.28%), and *Collinsella* (1.27%) had high abundance in both healthy and FLD groups (Figure 1B). 

To explore the impact of NAFLD on the children’s gut microbiota, we compared the microbiota of healthy children and children with NAFLD. Most alpha diversity indices were not significantly different between healthy and FLD groups (Supplementary Figure S2C,D). The PCoA plot shows significant differences in beta diversity between the healthy children and FLD groups, indicating the separation of microbial communities (Figure 1C). LEfSe analysis showed that Bacteroidetes, Verrucomicrobia, *Bacteroides*, *Peptoclostridium*, and *Akkermansia* were significantly abundant in the healthy than in the FLD group. In the FLD group, Actinobacteria, *Collinsella*, *Escherichia-Shigella*, *Roseburia*, and *Bifidobacterium* were outstanding (Figure 1D). In addition, the ratio of Firmicutes and Bacteroidetes in the healthy group was significantly higher than that in the FLD group (Figure 1E). We further assessed the correlation between clinical indicators and genus abundance. In the healthy group, we did not find that the high abundant dominant genera were significantly associated with clinical indicators (Supplementary Figure S2E). In the FLD group, *Collinsella* was positively associated with ALT, *Akkermansia* and *Bacteroides* were positively associated with TG, and *Peptoclostridium* was negatively associated with CHE (Supplementary Figure S2F). 

**Figure 2:**
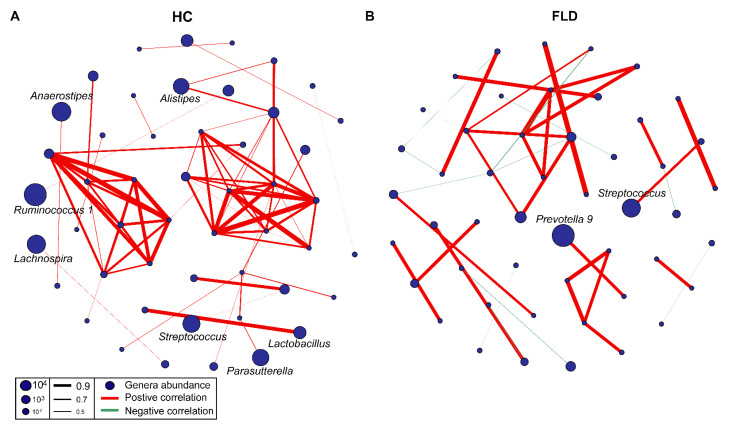
Microbial network comparison between children with NAFLD and healthy children. (**A**,**B**) The microbial network for children with NAFLD and healthy children.

Microbial co-occurrence networks can help us better understand the connections and interactions between genera. The microbial network of the healthy group was composed of 78 edges and 51 nodes. The abundances of *Anaerostipes*, *Ruminococcus 1*, *Lachnospira*, *Streptococcus*, *Parasutterella*, *Alistipes*, and *Lactobacillus* were high in the network. The interactions between genera were all positively correlated (Figure 2A). While the microbial network of the FLD group is relatively simple (39 edges, 49 nodes), only the abundance of *Prevotella 9* and *Streptococcus* were high. The interactions among genera were weak, and some genera were negatively correlated (positive correlation: 26 edges, negative correlation: 13 edges, Figure 2B). These microbial networks involve complex connections among multiple genera, and further comparisons are needed. We also found a similar trend in the correlation network between genus and clinical indicators. The genera were positively correlated with clinical indicators in the healthy group, such as RBC, WBC, NEUT, and IG. The network structure was relatively complex, with more edges and nodes (Supplementary Figure S2G). Interestingly, we found that the TG was positively correlated with several genera in the FLD group. The numbers of edges and nodes in the FLD group network were less than those in the healthy group network (Supplementary Figure S2H).

### 3.2. Gut Microbiota in Children with NAFLD Interplayed with Lymphocytes

**Figure 3:**
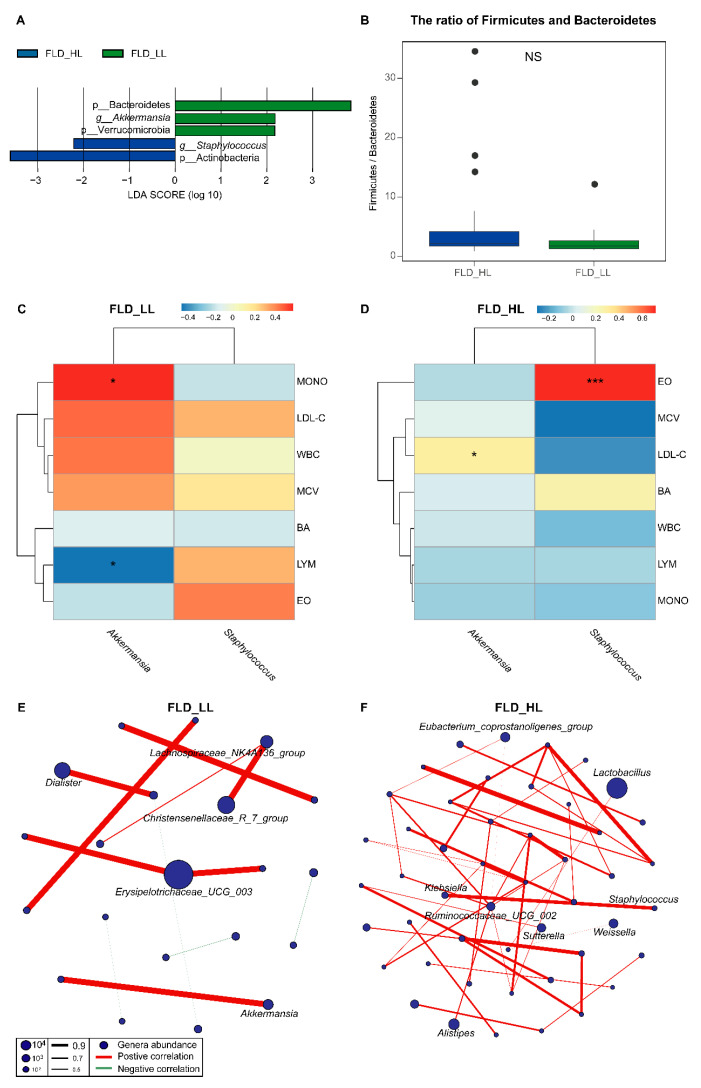
(**A**) Bacteroidetes, Verrucomicrobia, and *Akkermansia* were enriched in the FLD_LL group, while Actinobacteria and *Staphylococcus* were enriched in the FLD_HL group. (**B**) There was no difference in the ratio of Firmicutes and Bacteroidetes between FLD_HL and FLD_LL groups. (**C**) In the FLD_LL group, *Akkermansia* was positively correlated with MONO, and negatively correlated with LYM, * *p* < 0.05. (**D**) In the FLD_HL group, *Staphylococcus* and *Akkermansia* were positively correlated with EO and LDL-C, respectively, * *p* < 0.05, *** *p* < 0.001. (**E**,**F**) Microbial networks for FLD_HL and FLD_LL groups.

To understand the relationships among the NAFLD, gut microbiota, and lymphocytes, we compared gut microbiota differences between different lymphocyte counts of children with NAFLD. We further compared the gut microbiota of 41 children with NAFLD and high lymphocyte counts (LYM ≥3, FLD_HL group) and 22 children with NAFLD and low lymphocyte counts (LYM < 3, FLD_LL group). A number of studies have demonstrated that lymphocyte reference ranges are influenced by environmental factors and location [33,34]. The cutoff was chosen on the basis of the local reference range. FLD_HL group showed higher BA, EO, MONO, NEUT, WBC, and LYM, while LDL-C and MCV were higher in the FLD_LL group (Table 2 and Supplementary Figure S3). Alpha diversity analysis showed no significant difference in observed species and Shannon indices between FLD_HL and FLD_LL groups (Supplementary Figure S4A,B). The PCoA plot showed no significant difference in beta diversity between the two groups (Supplementary Figure S4C). LEfSe analysis revealed an apparent alteration of the microbiota characterized by higher Bacteroidetes, Verrucomicrobia, and *Akkermansia* in the FLD_LL groups. Actinobacteria and *Staphylococcus* were more abundant in the FLD_HL group (Figure 3A). However, the ratio of Firmicutes and Bacteroidetes between groups FLD_LL and FLD_HL was not statistically significant (Figure 3B).

We also calculated the correlation coefficient of clinical indicators and genus abundance. Figure 3C,D presents the results obtained from the correlation analysis. The abundance of *Akkermansia* had a positive association with MONO and LYM in the FLD_LL group (Figure 3C). In the FLD_HL group, *Staphylococcus* showed a significant positive correlation with EO, and *Akkermansia* was negatively correlated with LDL-C (Figure 3D). As displayed in Figure 3E,F, the numbers of nodes and edges in group FLD_HL (nodes: 48, edges: 46) were higher than those in group FLD_LL (nodes: 21, edges:12). The FLD_LL group network consisted of *Lachnospiraceae NK4A136 group*, *Dialister*, *Christensenellaceae R 7 group*, *Erysipelotrichaceae UCG 003*, and *Akkermansia* genera nodes (Figure 3E). *Eubacterium coprostanoligenes group*, *Ruminococcaceae UCG 002*, *Klebsiella*, *Staphylococcus*, *Sutterella*, *Weissella*, *Lactobacillus*, and *Alistipes* are the key nodes in the FLD_HL group network (Figure 3F). The clinical indicator and microbial network of FLD_LL and FLD_HL groups appeared relatively simple. WBC was positively associated with *Roseburia* and *Megamonas* in the FLD_LL group (Supplementary Figure S4D). EO was positively associated with *Klebsiella* and *Staphylococcus* in the FLD_HL group (Supplementary Figure S4E).

### 3.3. Differences in the Gut Microbiome between NAFLD and Healthy Children

**Figure 4:**
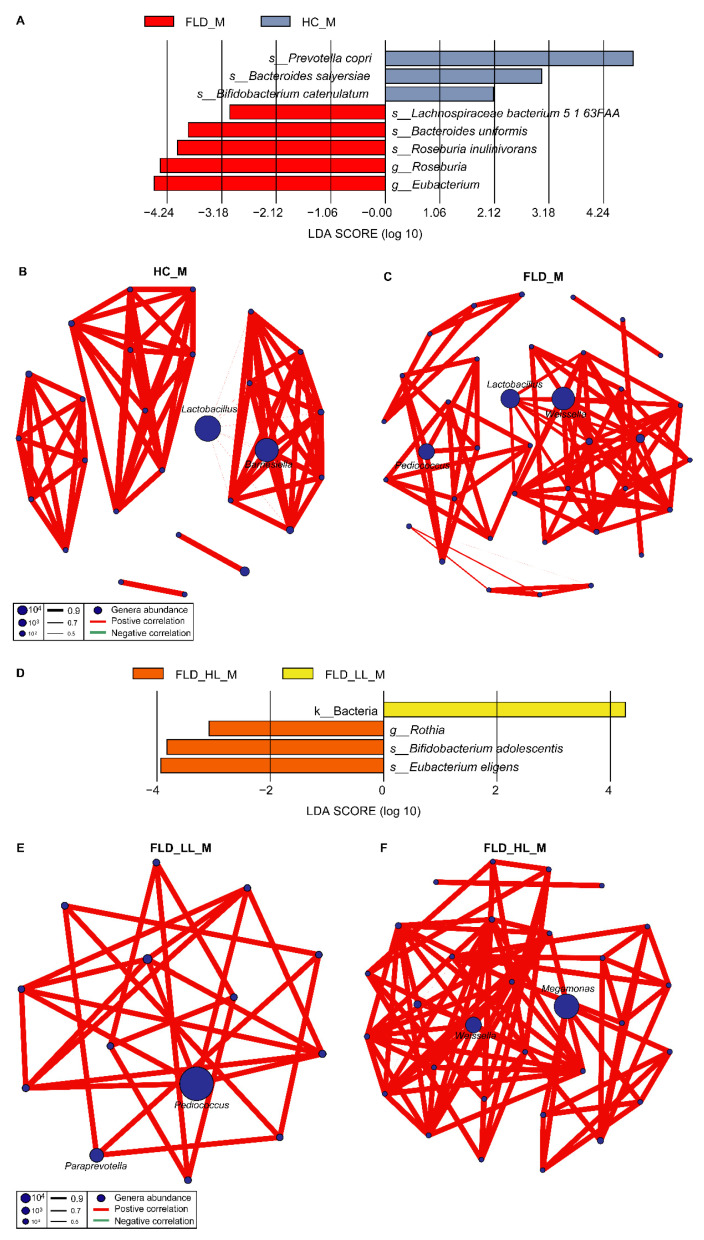
(**A**) The microbial biomarkers in groups HC_M and FLD_M include *Prevotella copri*, *Bacteroides salyersiae*, *Bifidobacterium catenulatum*, *Eubacterium*, *Roseburia*, *Lachnospiraceae bacterium 5 1 63FAA*, *Bacteroides uniformis*, and *Roseburia inulinivorans*. (**B**,**C**) Microbiome network of groups HC_M and FLD_M. (**D**) LEfSe analysis revealed that *Rothia*, *Eubacterium eligens*, and *Bifidobacterium adolescentis* were enriched in group FLD_HL_M. (**E**,**F**) Microbiome network of groups FLD_LL_M and FLD_HL_M.

To verify the 16S rRNA gene sequencing results, we randomly selected 13 fecal samples (eight children with NAFLD and five healthy children) for metagenomic sequencing. After quality control, a total of 558,344,758 clean reads (average of 42,549,596 sequences per sample) were obtained for further analysis. No alpha diversity difference was seen between the metagenome of healthy children (HC_M) and children with NAFLD (FLD_M) groups based on Simpson and evenness indices (Supplementary Figure S4F,G). According to the beta diversity analysis, no differences were observed between the two groups (Supplementary Figure S4H). LEfSe analysis showed that taxa such as *Prevotella copri*, *Bacteroides salyersiae*, and *Bifidobacterium catenulatum* were significantly enriched in group HC_M. At the same time, *Eubacterium*, *Roseburia*, *Lachnospiraceae bacterium 5 1 63FAA*, *Bacteroides uniformis*, and *Roseburia inulinivorans* were enriched in group FLD_M (Figure 4A). We found that the microbial network of healthy children consisted of 27 nodes and 81 edges and was mainly constructed around *Lactobacillus* and *Barnesiella* (Figure 4B). The microbial network of children with NAFLD (37 nodes and 89 edges) is mainly composed of *Lactobacillus, Weisseria and Pediococcus* (Figure 4C). Hence, the microbial network of group HC_M differed from that of group FLD_M. 

### 3.4. Relationships between NAFLD, Gut Microbiome, and Lymphocytes

We further compared the gut microbiome of three children with NAFLD and low lymphatic counts and five children with NAFLD and high lymphatic counts. On the basis of Simpson and evenness indices, no significant reduction was identified in alpha diversity of the metagenome of children with NAFLD and high numbers of lymphocytes (FLD_HL_M) compared with the children with NAFLD and low numbers of lymphocytes (FLD_LL_M) groups (Supplementary Figure S4I,J). Evaluation of beta diversity also showed no differences between FLD_HL_M and FLD_LL_M groups (Supplementary Figure S4K). The LEfSe analysis results revealed significant taxa enrichment in the FLD_HL_M group, such as *Rothia*, *Eubacterium eligens*, and *Bifidobacterium adolescentis* (Figure 4D). In the microbial network comparison of different groups, we observed that *Weissella* and *Megamonas* were essential genera of group FLD_HL_M, while *Paraprevotella* and *Pediococcus* were important genera in the FLD_LL_M group (Figure 4E). The network topology of group FLD_HL_M was different from that of group FLD_LL_M (FLD_LL_M: 14 nodes, 27 edges; FLD_HL_M: 30 nodes, 96 edges, Figure 4F). Moreover, it was interesting that the FLD_M network was similar to the combination of FLD_HL_M and FLD_LL_M networks. The hub genera (*Pediococcus* and *Weissella*) of the FLD_M network were divided into FLD_LL_M and FLD_HL_M networks and became the hub genera in the two networks, respectively.

## 4. Discussion

Our study found that lymphocyte counts disturb the association between the NAFLD and gut microbiota. We observed that NAFLD was associated with a decrease in the abundance of Bacteroidetes, Verrucomicrobia, and *Akkermansia*, and an increase in the abundance of *Actinobacteria* in children’s gut microbiota. Low lymphocyte blunts the association. The gut microbiota of children is disturbed by the influence of NAFLD, and various proinflammatory bacteria, such as *Collinsella*, *Escherichia-Shigella*, and *Bacteroides uniformis*, were found. High lymphocyte counts are associated with gut microbiota disturbances in children with NAFLD and are also associated with an abundance of *Rothia* and *Staphylococcus* opportunistic pathogens. Moreover, NAFLD is associated with children’s microbial networks and their complex interactions with clinical indicators. We observed that when children with NAFLD had comparatively low lymphocyte levels, the microbial network and its co-network with clinical indicators were similar to those in healthy children.

### 4.1. Alpha and Beta Diversity of Gut Microbiota in Children Is Less Affected by NAFLD

In our study, significant differences were observed only in beta diversity between healthy children and children with NAFLD, consistent with other studies [4]. There are few studies on the influence of lymphocyte level on the beta diversity of gut microbiota [10,35]. We did not find differences in beta diversity between the FLD_HL and FLD_LL groups. We speculate that this probably due to the fact that the participants in both FLD_HL and FLD_LL groups were all children with NAFLD. It may also be that the beta diversity of the gut microbiota in children with NAFLD is less affected by the lymphocytes. We saw a trend of NAFLD and lymphocyte influences on children’s gut microbiota, notably variations in microbial abundance and networks.

### 4.2. The Disturbance of Gut Microbiota Is Associated with NAFLD and Lymphocytes

We found that NAFLD was associated with a decreased abundance of Bacteroidetes, Verrucomicrobia and *Akkermansia*, and an increased abundance of Actinobacteria in children’s gut microbiota. Compared with FLD_HL group children, the FLD_LL group children with low lymphocyte levels had higher abundances of Bacteroidetes, Verrucomicrobia, and *Akkermansia*, and lower abundances of Actinobacteria. We speculate that low lymphocyte levels reverse the abundance of these phyla and genera in the gut microbiota of children with NAFLD. We also found that the abundance of *Akkermansia* was negatively correlated with lymphocytes. Verrucobacterium and *Akkermansia* directly reverse the disease phenotype of obesity by regulating intestine barrier function, lipid metabolism, and glucose homeostasis in obese mice [36,37]. *Akkermansia* also inhibits lymphocyte proliferation and maintains gut mucosal barrier integrity and microbiota homeostasis by activating the Toll-like receptor 2 signaling pathway [38]. We believe that Verrucobacterium and *Akkermansia* inhibit lymphocyte proliferation and suppress the development of NAFLD by regulating lipid metabolism and glucose homeostasis.

When incubated together with murine lymphocytes, Bacteroidetes has a dose-dependent inhibition of the blastogenic transformation of lymphocytes stimulated by *Escherichia coli* lipopolysaccharide or concanavalin and, therefore, affected T-cell-dependent immunity [39]. The presence of a high abundance of alcohol-producing bacteria Actinobacteria is seen in the intestines of NAFLD patients [40]. Actinobacteria was negatively correlated with tight junction proteins and positively correlated with enhanced expression of proinflammatory cytokines [41]. A varied trend of microbial abundance was seen in the 16S rRNA gene results. These results suggest that the NAFLD may disturb gut microbiota in children, and low lymphocyte levels may modulate this disturbance. 

Children with NAFLD showed low abundance genera, including *Bacteroides* and *Peptoclostridium*. However, an abundance of proinflammatory microbes *Collinsella* and *Escherichia-Shigella* was noted in children with NAFLD. *Collinsella* is positively associated with ALT in the gut microbiota of children with NAFLD. *Staphylococcus* was enriched in the FLD_HL group. *Bacteroides* suppresses the inflammatory response of NAFLD disease by modulating the immune system, increasing HDL, and reducing ALT [40]. *Peptoclostridium* is considered a commensal organism in an immunocompetent host, suppressing opportunistic pathogens and inflammatory responses [42]. *Collinsella*, a 7alpha-dehydroxylated bacterium, induces an inflammatory response and contributes to NAFLD development by promoting secondary bile acid production and elevating gut permeability [43]. The majority of the genes related to lipopolysaccharide biosynthesis have been observed in *Escherichia-Shigella* [44]. The human innate immunity is stimulated by peptidoglycan of *Staphylococcus*, which induces cytokines and antibacterial peptides to promote lymphocyte proliferation [45]. Our study suggests that NAFLD may contribute to a dysbiosis of the gut microbiota in children, with increased abundances of *Collinsella* and *Escherichia-Shigella* proinflammatory bacteria. High lymphocyte counts further promote inflammatory responses and increase the abundance of *Staphylococcus* opportunistic pathogens in children with NAFLD. Together, we consider that Bacteroidetes, *Bacteroides*, and *Peptoclostridium* suppress the inflammatory response and alleviate NAFLD by regulating the immune system. Actinobacteria, *Collinsella*, and *Escherichia-Shigella* induce proinflammatory factor expression, promote inflammation, and worsen NAFLD.

### 4.3. The Interplay among NAFLD, Gut Microbiome, and Lymphocytes

Notable biomarkers of the HC_M group were *Prevotella copri*, *Bifidobacterium catenulatum*, and *Bacteroides salyersiae*, while *Roseburia inulinivorans* and *Bacteroides uniformis* were biomarkers in the FLD_M group. *Rothia* were enriched in the FLD_HL_M group. *Prevotella copri* reduces lymphocyte infiltration and prevents inflammatory responses by activating Toll-like receptor 2 [46]. *Bifidobacterium catenulatum* is anti-inflammatory and contributes to other probiotics that produce butyrate [47]. *Roseburia inulinivorans* carries a gene encoding proinflammatory flagellin that induces interleukin-8 and promotes the aggregation of immune cells, leading to the inflammatory response [48]. *Bacteroides uniformis* was significantly positively correlated with lipopolysaccharide and triglyceride concentrations and was associated with inflammation [49]. *Rothia* is considered to be an opportunistic pathogen and has also been observed in the intestines of patients with alcoholic liver disease, inducing microbial dysbiosis and elevated microbial translocation and resulting in a more vigorous immune and inflammatory response [50]. We speculate that a combination of NAFLD and high lymphocyte levels may result in *Bacteroides uniformis* proinflammatory bacteria being found in children’s gut microbiota. High levels of lymphocytes maintain the gut microenvironment in an inflammatory state and promote the increased abundance of the *Rothia* opportunistic pathogen. We found no consistent genus or species in 16S rRNA gene and metagenomic results. However, we observed that NAFLD disrupts gut microbiota in children, mitigated somewhat by low levels of lymphocytes, while higher levels of lymphocytes may lead to more severe inflammation and dysbiosis. In short, *Prevotella copri* and *Bifidobacterium catenulatum* promote short-chain fatty-acid production, inhibit lymphocyte infiltration and inflammation, and alleviate NAFLD. *Roseburia inulinivorans*, *Bacteroides uniformis* and *Rothia* induce microbiota disorders and immune responses that lead to more severe NAFLD.

### 4.4. NAFLD Alters Gut Microbiota Microbial Networks in Children

Microbial networks are more sensitive to external stimuli than microbial community composition [45]. We found that the interactions between genera were positively correlated in the microbial network topology of healthy children. Conversely, a negative correlation between the genera was found in the microbial network of children with NAFLD. Positive correlations between selected bacteria and genera in a microbial network help maintain a stable network structure [51]. The stable microbial network structure enables the network against the invasion of the undesirable external stimulation and contributes to the body maintaining normal homeostasis [52]. In contrast, the microorganisms of inflammatory bowel disease patients have inhibitory effects on each other, and the microbial network structure is more fragile [53]. We conclude that NAFLD is associated with analogous changes in the gut microbial network in children.

Our study showed that the microbial network structure of group FLD_HL was different from that of group FLD_LL. We observed that *Lachnospiraceae NK4A136 group*, *Dialister*, *Christensenellaceae R 7 group*, *Erysipelotrichaceae UCG 003*, and *Akkermansia* were more abundant in FLD_LL group network. The FLD_HL network consists of *Eubacterium coprostanoligenes group*, *Ruminococcaceae UCG 002*, *Klebsiella*, *Staphylococcus*, *Sutterella*, *Weissella*, *Lactobacillus*, and *Alistipes*. *Erysipelotrichaceae UCG 003* belongs to the *Erysipelotrichaceae*. *Erysipelotrichaceae* were found to be enriched in the intestine of obese patients [54]. *Lachnospiraceae NK4A136 group* is a butyrate-producing bacteria that exerts an inflammation-suppressing effect in human gut mucosa [55]. The *Christensenellaceae R 7 group* ferments proteins to butyrate. Butyrate can repair gut mucosal damage, increase the expression of ZO-1 protein, improve the gut barrier capacity, and reduce the level of gut endotoxin [56]. Taken together, *Erysipelotrichaceae UCG 003*, *Lachnospiraceae NK4A136 group*, and *Christensenellaceae R 7 group* contribute to the alleviation of NAFLD in children by reducing damage of the intestinal barrier, reducing intestinal endotoxin levels, and suppressing inflammatory responses.

Jiang’s study also found that the relative abundance of *Lactobacillus* was increased in NAFLD patients compared to healthy subject [57]. *Alistipes* is considered to be a specific biomarker of oxidative damage to hepatic cells, fibrosis, and gut microbiota disturbances in NAFLD patients [58]. *Eubacterium coprostanoligenes group* and *Ruminococcaceae UCG 002* belong to Ruminococcaceae, which was thought to promote hepatocyte fibrosis in NAFLD patients [59]. We believed that low lymphocyte levels suppress inflammation and microbial network disturbances in children with NAFLD through multiple pathways. A high level of lymphocytes might aggravate the disorder of the microbial network in children with NAFLD. *Alistipes*, *Eubacterium coprostanoligenes group*, and *Ruminococcaceae UCG 002* increase liver fibrosis and lead to further hepatocyte damage.

### 4.5. NAFLD Alters Gut Microbiome Microbial Networks in Children

According to metagenomic sequencing to elucidate the gut microbiome of children with NAFLD, *Barnesiell* is the key node of the microbial networks of group HC_M. *Weissella* and *Pediococcus* are the important nodes of the microbial networks of group FLD_M. *Barnesiella* has close contact with immune cells and exerts anti-inflammatory protection [60]. *Weissella* may convert glucose and fructose directly into ethanol in the rat intestine, increasing ethanol production [61]. It results in hyperlipidemia and may promote the development of NAFLD [61]. *Pediococcus* is a probiotic that improves NAFLD by modulating gut microbiome and inflammatory responses [62]. 

Both anti- and proinflammatory genera are present in the gut microbiome of children with NAFLD. However, the network comparison results of groups FLD_HL_M and FLD_LL_M may help elucidate this phenomenon. The hub genera *Weissella* and *Pediococcus* of the FLD_M network were divided into FLD_HL_M and FLD_LL_M networks and became the hub genera in the two networks, respectively. The main nodes *Barnesiella* and *Pediococcus* in the HC_M and FLD_LL_M networks can both play a role in suppressing inflammation; thus, we think that HC_M and FLD_LL_M networks are functionally similar. Therefore, we propose that NAFLD and lymphocyte numbers affect children’s gut microbiome network. We hypothesize that low lymphocyte numbers might alter the network function of children with NAFLD to resemble those of healthy children.

### 4.6. Impact of NAFLD on the Microbial Network of Clinical Indicators in Children

We found that WBC was the critical node in the clinical indicators and microbial network of the healthy group, and TG was the critical node in the FLD network. The ratio of Firmicutes and Bacteroidetes in the FLD group was higher than that in the healthy group. Accumulation of TG over 5% of the total liver weight is characteristic of NAFLD [63]. We speculate that TG may be a key node in the clinical indicator microbial network of children with NAFLD due to the NAFLD itself. Another possibility is that the level of TG in children with NAFLD is higher, and the gut microbiota is involved in the metabolic process of TG, resulting in the close relationship between the gut microbiota and TG in children with NAFLD.

### 4.7. Influence of Lymphocytes on the Microbial Network of Clinical Indicators in Children with NAFLD

Consistent with previous microbial network trends, the WBC node was found in both clinical indicators and microbial network of FLD_LL and healthy groups. We discovered that *Klebsiella*, *Staphylococcus* and EO were positively correlated in the FLD_HL group. Similarly, we found that *Klebsiella* and *Staphylococcus* pathogens were positively associated with EO. The impact of lipoteichoic acid significantly increased the number of eosinophils in local tissues in *Staphylococcus* [64]. Metabolites of *Klebsiella* induce an increase in reactive oxygen species and total superoxide dismutase activity, increasing eosinophil numbers [65]. We speculate that groups of FLD_LL and healthy have the same WBC nodes, possibly because the progression of NAFLD is inhibited by low lymphocyte levels. The presence of opportunistic pathogens *Staphylococcus*, *Klebsiella*, and eosinophils in the FLD_HL group may be due to the high lymphocyte count indicating a more severe inflammatory state. 

Our study had limitations. Our cross-sectional observation in gut microbiota in children with NAFLD and elevated lymphocytes may be transient. We have not yet identified specific lymphocyte subsets that play a major role in the relationship between NAFLD and gut microbiota. We hope to elucidate remaining questions in the ongoing prospective cohort study.

## 5. Conclusions

Our study revealed the changing trends of gut microbiota by exploring the relationship among NAFLD, gut microbiota, and lymphocytes. The microbial abundance in children may be disturbed by NAFLD, leading to the emergence of proinflammatory bacteria, while low levels of lymphocytes may attenuate this disturbance. The gut microbial network of children with NAFLD and low levels of lymphocytes seems to be structurally and/or functionally similar to healthy children. These findings provide hints for understanding the relationship between pediatric NAFLD, gut microbiota, and lymphocytes. Multi-omics approaches may shed light in this regard in the future.

## Figures and Tables

**Table 1:** Differences in clinical indicators of NAFLD and healthy children.

Parameters	HC (n = 63)		FLD (n = 63)		*t*/*χ*^2^/*Z*	*p*-Value
	$\bar{x}$ ± SD	M (q1, q3)	$\bar{x}$ ± SD	M (q1, q3)		
Boy (n, %) ^1^	47 (74.60)		47 (74.60)		<0.01	1
Age (years) ^3^	6.72 ± 0.32	6.73 (6.44, 7.02)	6.75 ± 0.31	6.71 (6.55, 6.93)	−0.32	0.75
Height (cm) ^2^	122.90 ± 4.81	122.45 (119.78, 125.75)	127.72 ± 4.45	127.6 (124.4, 131)	−5.84	*p* < 0.001
Waist (cm) ^2^	55.79 ± 5.13	55.3 (52.31, 58.35)	71.18 ± 8.35	70.5 (65.2, 77.3)	−12.44	*p* < 0.001
Hip (cm) ^2^	67.16 ± 5.32	67.95 (62.64, 70.51)	79.41 ± 5.84	78.75 (75.38, 82.88)	−12.27	*p* < 0.001
Weight (kg) ^2^	24.39 ± 3.64	24.5 (21.75, 26.8)	36.03 ± 6.40	34.7 (32.15, 39.5)	−12.56	*p* < 0.001
BMI (kg/m^2^) ^3^	16.07 ± 1.62	16.46 (14.73, 17.42)	21.98 ± 3.03	21.54 (20.19, 23.91)	−9.13	*p* < 0.001
PBF (%) ^2^	20.13 ± 6.02	19.5 (15.25, 23.8)	36.05 ± 6.23	36.7 (32.9, 39)	−14.58	*p* < 0.001
VFA (cm^2^) ^3^	20.81 ± 8.44	18 (15.65, 24.75)	66.06 ± 30.13	64.9 (46.2, 82.6)	−8.66	*p* < 0.001

VFA: visceral fat area, PBF: percentage of body fat, BMI: body mass index. There was one missing value for each of the waist and hip indicators in the HC group. ^1^: Chi-square tests, *χ*^2^ value; ^2^: independent *t*-tests, *t* value; ^3^: Wilcoxon rank-sum tests, *Z* value.

**Table 2:** Comparison of clinical indicators between groups FLD_HL and FLD_LL.

Parameters	FLD_HL (n = 41)		FLD_LL (n = 22)		*t*/*χ*^2^/*Z*	*p*-Value
	$\bar{x}$ ± SD	M (q1, q3)	$\bar{x}$ ± SD	M (q1, q3)		
Boy (n, %) ^1^	33 (80.49)		14 (63.64)		2.15	0.143
Age (years) ^2^	6.72 ± 0.30	6.71 (6.46, 6.9)	6.79 ± 0.32	6.73 (6.6, 6.96)	−0.74	0.462
Height (cm) ^2^	127.57 ± 4.53	127.3 (124.35, 129.25)	127.99 ± 4.39	127.68 (124.85, 131.11)	−0.35	0.725
Waist (cm) ^2^	71.27 ± 8.01	70.25 (66.75, 77.35)	71.01 ± 9.14	70.88 (63.98, 74.21)	0.11	0.909
Hip (cm) ^2^	78.92 ± 5.79	78.75 (74.7, 82.75)	80.32 ± 5.94	79.9 (77.06, 82.41)	−0.9	0.371
Weight (kg) ^2^	35.84 ± 6.05	34.6 (32.7, 39.8)	36.40 ± 7.13	35.6 (31.28, 38.98)	−0.31	0.755
BMI (kg/m^2^) ^3^	21.89 ± 2.63	21.57 (20.41, 24.18)	22.14 ± 3.73	21.13 (19.92, 23.36)	−0.45	0.65
PBF (%) ^2^	35.73 ± 6.27	36.8 (33.1, 38.4)	36.65 ± 6.27	36.35 (32.85, 40.38)	−0.55	0.583
VFA (cm^2^) ^3^	64.52 ± 28.72	65.8 (46.1, 82.4)	68.92 ± 33.10	61.85 (48.5, 82.4)	−0.08	0.937

VFA: visceral fat area, PBF: percentage of body fat, BMI: body mass index. ^1^: Chi-square tests, *χ*^2^ value; ^2^: independent *t*-tests, *t* value; ^3^: Wilcoxon rank-sum tests, *Z* value.

## Data Availability

The raw sequence data were deposited in the Genome Sequence Archive in Beijing Institute of Genomics Data Center with accession numbers CRA007292 and CRA007303. The shared URL is http://bigd.big.ac.cn, accessed on 2 November 2022.

## Supplementary Materials

The following supporting information can be downloaded at https://www.mdpi.com/article/10.3390/nu14214641/s1: Figure S1. The clinical indicators of WBC, RBC, HGB, HCT, MONO, NEUT, IG, TG, ALP, CHE, GLB, TP, ALT, GGT, and PA were higher in children with NAFLD, while HDL-C was higher in healthy children; * *p* < 0.05, ** *p* < 0.01, *** *p* < 0.001, and **** *p* < 0.0001; Figure S2. (A,B) Rank abundance and rarefaction curves of bacterial OTUs. (C,D) No difference in gut microbiota alpha diversity were observed between healthy children and children with NAFLD (*p* > 0.05). (E) No clinical indicators were significantly associated with genus abundance in the healthy group. (F) In the FLD group, *Collinsella* was positively correlated with ALT, TG was positively correlated with *Akkermansia* and *Bacteroides*, and *Peptoclostridium* was negatively correlated with CHE; * *p* < 0.05, ** *p* < 0.01, and *** *p* < 0.001. (G,H) The clinical indicators and microbial network for children with NAFLD and healthy children; Figure S3. EO, BA, MONO, NEUT, WBC, and LYM were higher in the FLD_HL group, while LDL-C and MCV were higher in the FLD_LL group; * *p* < 0.05, ** *p* < 0.01, and **** *p* < 0.0001; Figure S4. No differences in alpha (A,B) or beta diversity (C) in the microbial communities between FLD_HL and FLD_LL groups were observed (R^2^ = 0.021, *p* > 0.05). (D,E) The clinical indicators and microbial network in groups FLD_HL and FLD_LL. (F–H) There was no significant difference in alpha and beta diversity on the boxplot or PCoA plots between HC_M and FLD_M groups (R^2^ = 0.111, *p* > 0.05). (I–K) There was no difference in alpha and beta diversity between FLD_HL_M and FLD_LL_M groups (R^2^ = 0.171, *p* > 0.05).
