# Supplementary figures and images for: Interplay of Lymphocytes with the Intestinal Microbiota in Children with Nonalcoholic Fatty Liver Disease
Source: Nutrients. 2022 Nov 3;14(21):4641. doi: 10.3390/nu14214641 (PMC9657134; doi:10.3390/nu14214641)

Figure S1

Group FLD HC

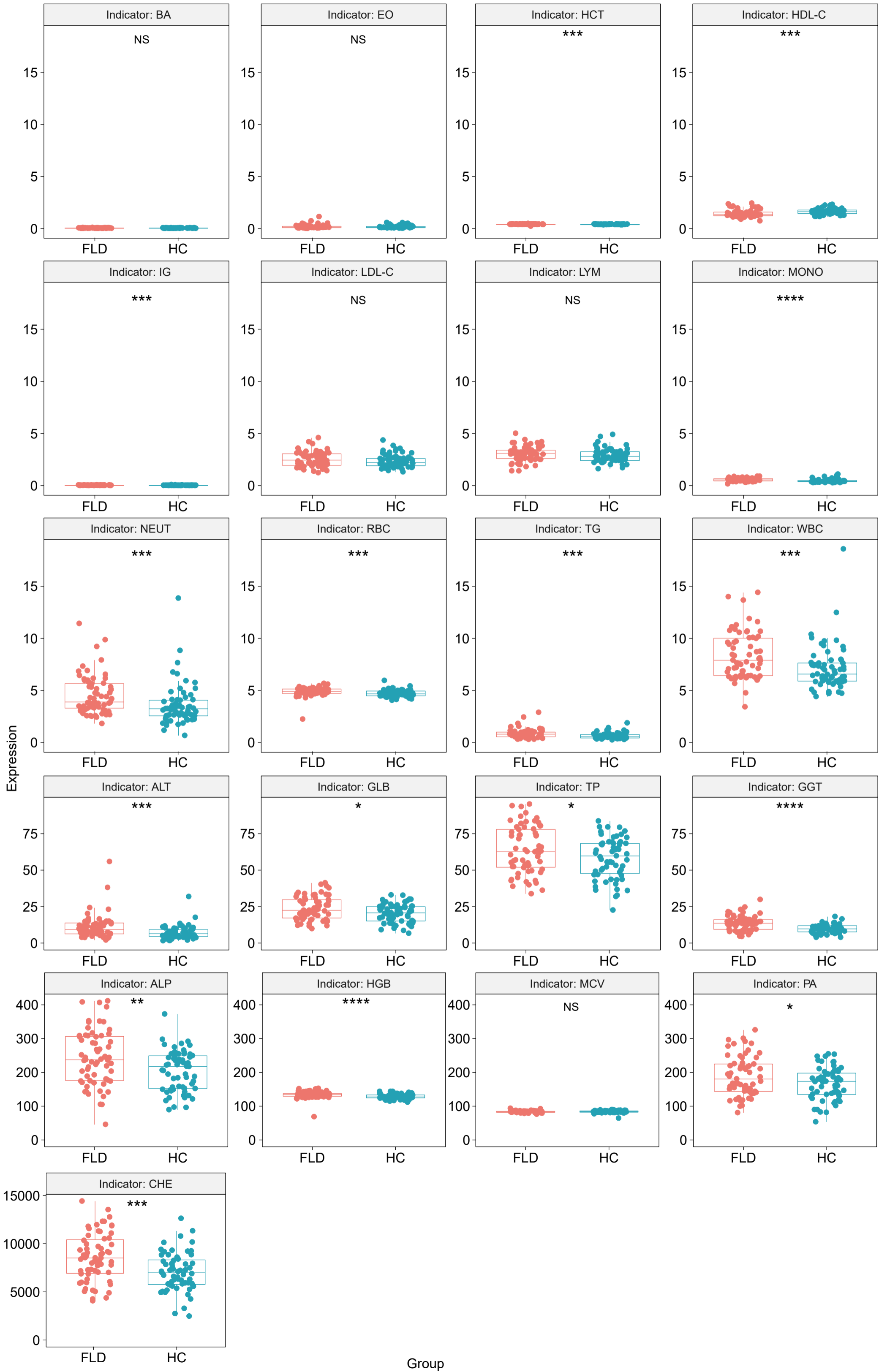

Supplement: Supplementary file 1 [file nutrients-14-04641-s001.zip › Figure S1.pdf]

Figure S2

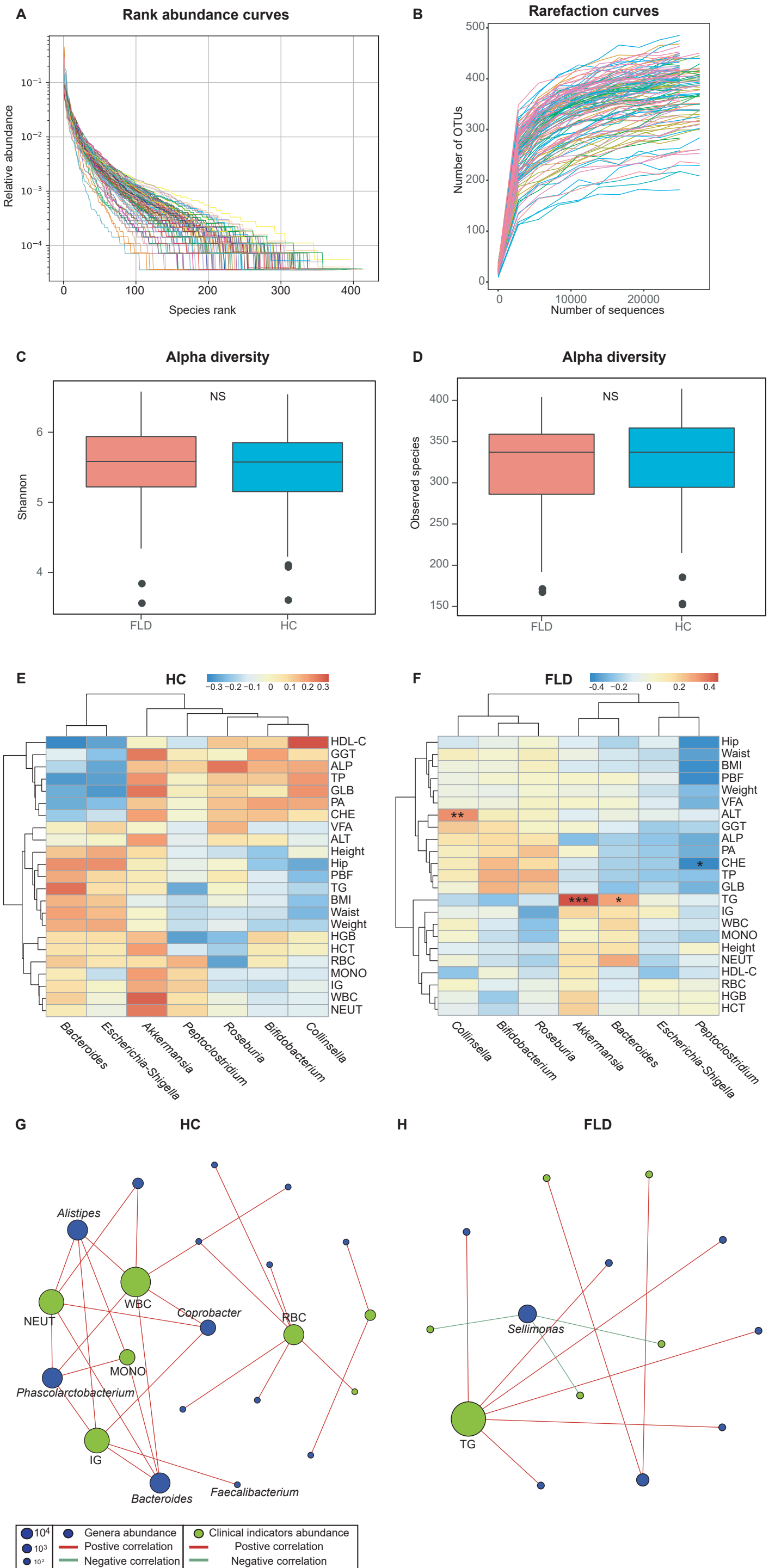

Supplement: Supplementary file 1 [file nutrients-14-04641-s001.zip › Figure S2.pdf]

Figure S3

Group FLD\_HL FLD\_LL

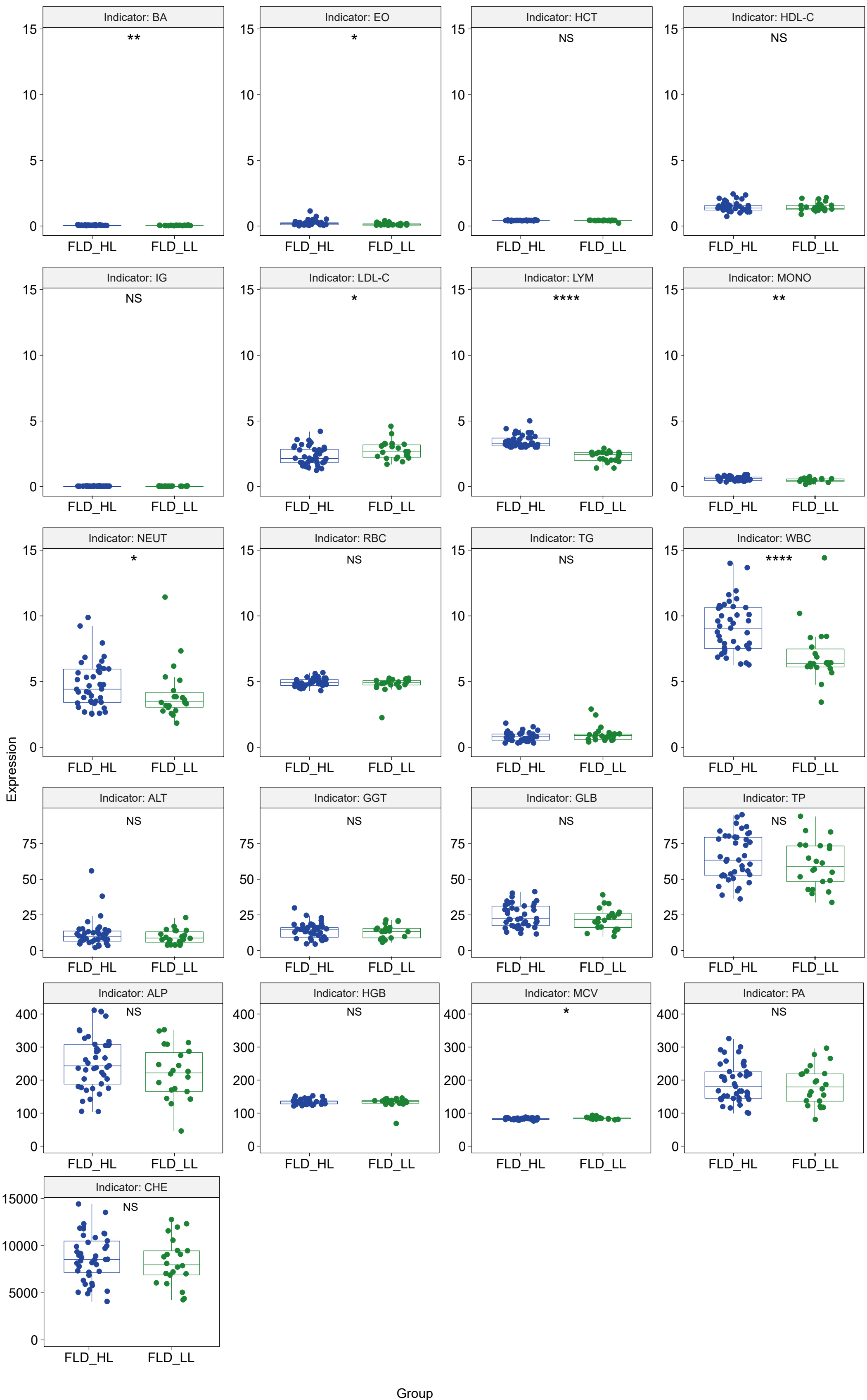

Supplement: Supplementary file 1 [file nutrients-14-04641-s001.zip › Figure S3.pdf]

Figure S4

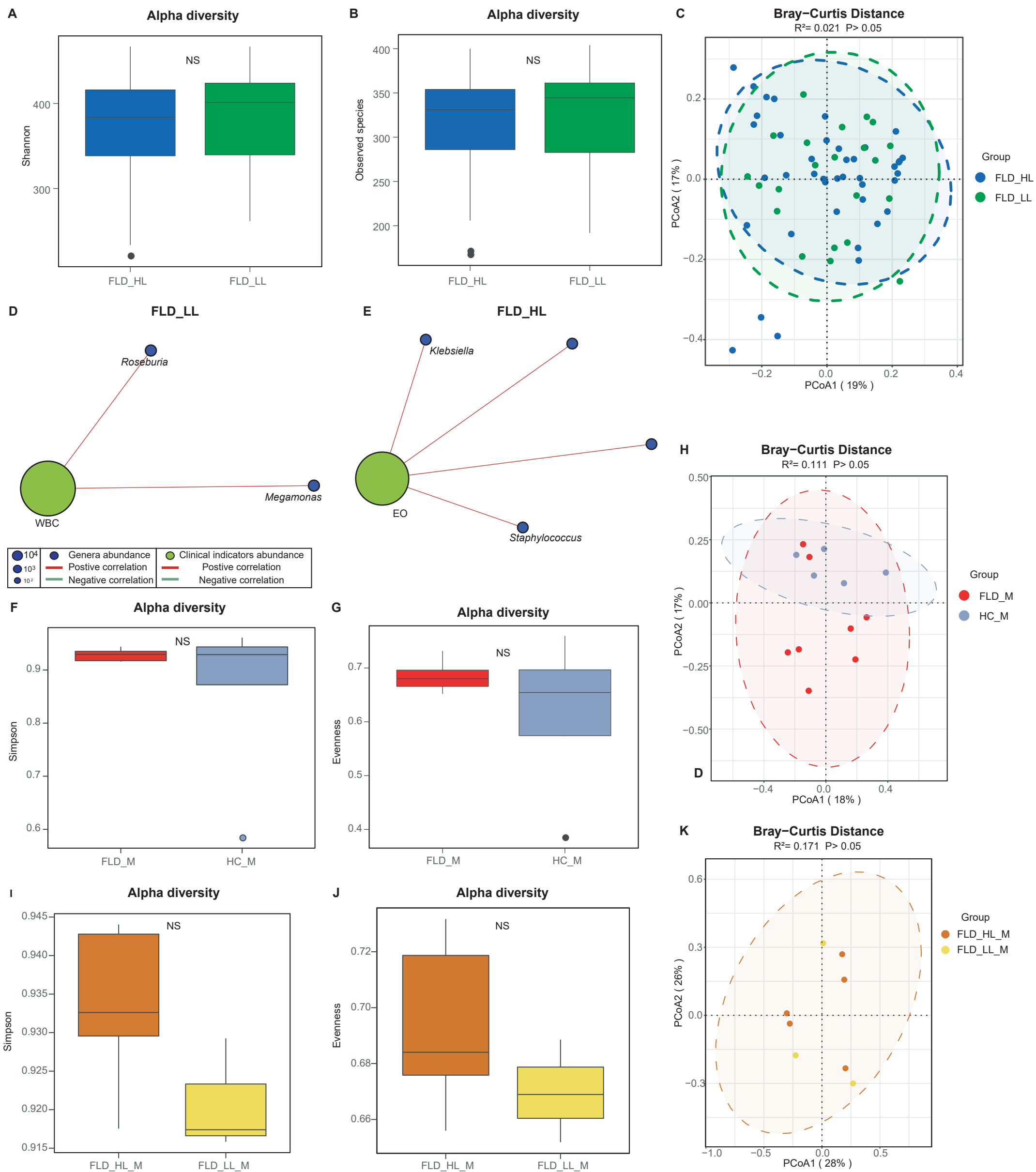

Supplement: Supplementary file 1 [file nutrients-14-04641-s001.zip › Figure S4.pdf]
